# Wetland fishes avoid a carbon dioxide deterrent deployed in the field

**DOI:** 10.1093/conphys/coac021

**Published:** 2022-05-06

**Authors:** P A Bzonek, N E Mandrak

**Affiliations:** Department of Ecology and Evolutionary Biology, University of Toronto, 27 King's College Circle, Toronto, Ontario, M5S 1A1, Canada; Department of Biological Sciences, University of Toronto Scarborough 1265 Military Trail, Scarborough, Ontario, M1C 1A4, Canada; Department of Ecology and Evolutionary Biology, University of Toronto, 27 King's College Circle, Toronto, Ontario, M5S 1A1, Canada; Department of Biological Sciences, University of Toronto Scarborough 1265 Military Trail, Scarborough, Ontario, M1C 1A4, Canada

**Keywords:** non-structural deterrent, non-physical barrier, invasive species, fishway, Cyprinus carpio, common carp, Carbon dioxide

## Abstract

Biological invasions are poorly controlled and contribute to the loss of ecosystem services and function. Altered watershed connectivity contributes to aquatic invasions, but such hydrologic connections have become important for human transport. Carbon dioxide (CO_2_) deterrents have been proposed to control the range expansion of invasive fishes, particularly through altered hydrologic connections, without impeding human transport. However, the effectiveness of CO_2_ deterrents needs to be further evaluated in the field, where fishes are situated in their natural environment and logistical challenges are present.

We deployed a proof-of-concept CO_2_ deterrent within a trap-and-sort fishway in Cootes Paradise, Ontario, Canada, to determine the avoidance responses of fishes attempting to disperse into a wetland. We aimed to describe deterrent efficiency for our target species, common carp, and for native fishes dispersing into the wetland. Our inexpensive inline CO_2_ deterrent was deployed quickly and rapidly produced a CO_2_ plume of 60 mg/l. Over 2000 fishes, representing 13 species, were captured between 23 May and 8 July 2019. A generalized linear model determined that the catch rates of our target species, common carp (*n* = 1662), decreased significantly during deterrent activation, with catch rates falling from 2.56 to 0.26 individuals per hour. Aggregated catch rates for low-abundance species (*n* < 150 individuals per species) also decreased, while catch rates for non-target brown bullhead (*n* = 294) increased. Species did not express a phylogenetic signal in avoidance responses. These results indicate that CO_2_ deterrents produce a robust common carp avoidance response in the field. This pilot study deployed an inexpensive and rapidly operating deterrent, but to be a reliable management tool, permanent deterrents would need to produce a more concentrated CO_2_ plume with greater infrastructural support.

## Teaser

We deployed a CO_2_ deterrent in the field and captured over 2000 fishes representing 13 species. The deterrent reduced catch rates for all species except brown bullhead. Common carp catch rates decreased 10-fold. These results indicate that an inexpensive and rapidly operating deterrent can produce robust responses in the field.

## Introduction

Human activities have drastically altered the biodiversity of freshwater systems (Su *et al.,* 2021). Threats, such as biological invasions, are poorly controlled (Strayer, 2010), but contribute to the loss of ecosystem diversity and resilience (Ricciardi and MacIsaac, 2011; Downing *et al.,* 2012). Freshwater ecosystems experience high invasion rates due to ballast-water transport, authorized and unauthorized release and altered system connectivity (Strayer, 2010). While the construction of dams have restricted access to critical habitats (Milt *et al.,* 2018), canals have allowed for the hydrologic connection of historically separated basins (Strayer, 2010; Rasmussen *et al.,* 2011).

Non-structural deterrents have been proposed to control the range expansion of invasive fishes without altering human transport. Non-structural deterrents can alter fish behaviour to produce avoidance responses as documented for acoustic, visual or low-concentration CO_2_ deterrents (Vetter *et al.,* 2015; Cupp *et al.,* 2016; Dennis *et al.,* 2019; Putland and Mensinger, 2019; Dennis and Sorensen, 2020), or they can alter fish physiology to limit the capacity to move, as documented for electrical (Noatch and Suski, 2012) and higher concentration CO_2_ (Suski, 2020) deterrents. Many fish species detect CO_2_ concentrations through chemoreceptors in their gills (Gilmour, 2001) and use this information to avoid suboptimal, hypercapnic environments (Kates *et al.,* 2012; Donaldson *et al.,* 2016; Suski, 2020). Increased CO_2_ concentrations are often associated with decreased O_2_ concentrations, but can also affect gas exchange at the gills and induce hyperventilation (Gilmour, 2001; Perry and Gilmour, 2002).

Recent deterrent efforts have been applied towards limiting the continued dispersal of invasive bigheaded carps (*Hypothalmichthys* spp.) within the Mississippi river basin (Kates *et al.,* 2012; Donaldson *et al.,* 2016). However, most CO_2_ deterrent studies have been conducted in the laboratory (see Kates *et al.,* 2012; Dennis *et al.,* 2015; Dennis *et al.,* 2016; Cupp *et al.,* 2017; Tucker *et al.,* 2018) or within artificial environments (see Donaldson *et al.,* 2016; Cupp*et al.,* 2016; Schneider *et al.,* 2018). There remains a paucity of information on CO_2_ deterrent performance within realistic site conditions (but see Cupp *et al.,* 2018). Field deterrents present challenges that do not exist in the laboratory such as inclement weather and suboptimal infrastructure. Deterrents must also produce an avoidance response in fishes that exist within their natural contexts, but behaviours in the laboratory and field can vary widely (Calisi and Bentley, 2009). Field tests are needed to describe *in situ* CO_2_ deterrent efficiency. Furthermore, deterrent efficiencies should be described for both target species and native fishes likely to be affected by deterrent deployment.

We deployed a proof-of-concept CO_2_ deterrent within a trap-and-sort fishway in Cootes Paradise, Ontario, Canada. This same fishway was previously outfitted with an acoustic fish deterrent (Bzonek *et al.,* 2021), allowing for a comparison of deterrent technology. Cootes Paradise is a 250-ha drowned river-mouth marsh and a significant nursery for Lake Ontario fishes (Thomasen and Chow-Fraser, 2012). The fishway, in operation since 1997, was constructed to exclude invasive common carp (*Cyprinus carpio*) from the marsh.

Common carp is a globally invasive species with high fecundity and broad biotic and abiotic tolerances (Weber and Brown, 2009). Introduced common carp can cause ecosystem-level changes by increasing sediment mixing and reducing aquatic vegetation through benthic foraging (Bajer and Sorensen, 2014; Huser *et al.,* 2016). In Cootes Paradise, common carp had dominated the community prior to the installation of the fishway. It had comprised 90% of the fish community biomass (Lougheed *et al.,* 2004) and increased turbidity by 45% (Lougheed *et al.,* 1998).

We aimed to determine whether the CO_2_ deterrent was effective at reducing the catch rates of fishes attempting to disperse into the wetland. The catch rates of the target species, common carp, and all other fishes were recorded across alternating deterrent and control trials. We predicted that the CO_2_ deterrent would reduce the catch rate of all regularly encountered fish species.

## Methods

To quantify the effectiveness of a non-structural CO_2_ deterrent, the catch rates of fishes entering the fishway under ambient conditions were compared to the catch rates of fishes entering fishway during the deterrent treatment. The Royal Botanical Gardens fishway is a physical structure that separates the Cootes Paradise wetland from Hamilton Harbour at the western end of Lake Ontario (43°16′47.0′′N 79°53′35.0′′W). Native and non-native fishes regularly challenge the fishway to gain access into the wetland. The fishway blocks the outlet of Cootes Paradise with metal grating (spacing, 5.4–6.2 cm), such that small fishes can pass through the grating but large fishes wider than the grating spacing are funnelled towards six traps (four active) to be manually processed. For a detailed description of fishway operation, see (Bzonek *et al.,* 2021).

Traps were operated between 23 May and 8 July 2019; however, deterrent trials (*n* = 7) were not operated between 31 May and 24 June due to logistical constraints (i.e. flooding). Lake Ontario experienced record-high water levels in summer 2019 (Orr, 2019), which flooded the only access road to the Royal Botanical Gardens fishway and prevented CO_2_ dewar gas delivery.

**Figure 1:**
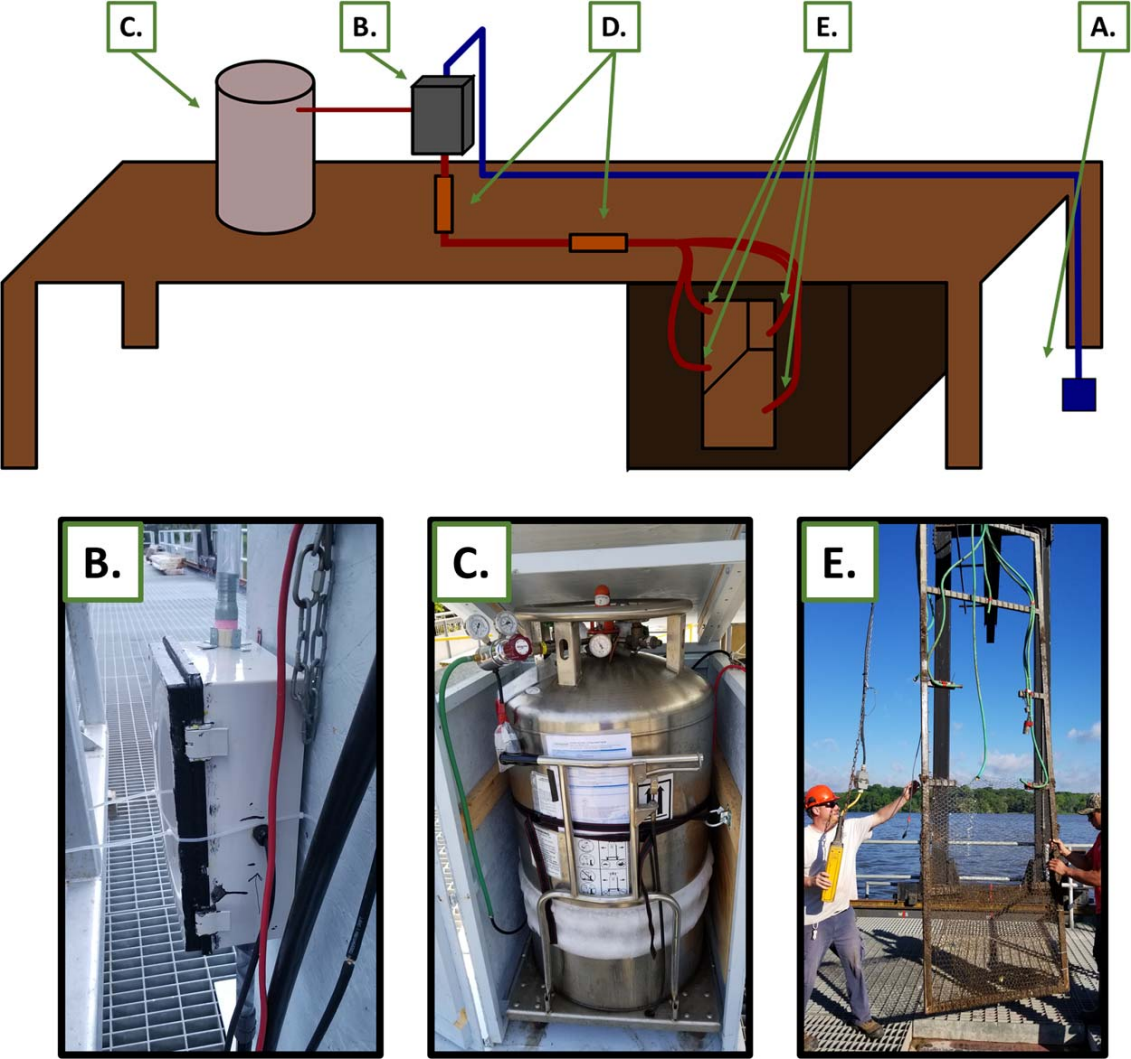
Schematic diagram and images of CO_2_ deterrent deployed at the Royal Botanical Gardens fishway, Ontario, Canada. A submersible pump (**A**) moved wetland water through polyethylene pipe into an injection manifold (**B**). CO_2_ gas was pumped from a liquid CO_2_ dewar (**C**) into the injection manifold (B) where it mixed with ambient wetland water. CO_2_ and water mixed throughout the manifold and hose delivery system, producing a stream of carbonated water. The mixing of CO_2_ and water was assisted with two static mixers (**D**). The carbonated water was introduced to a fishway trap at four locations (**E**) spanning the full depth of the trap opening.

The CO_2_ deterrent was installed in one of four active fishway traps and activated on alternating days, typically Monday, Wednesday and Friday. The traps were typically processed twice a day, Monday–Friday, at approximately 0800 and 1400 h with trap exposure time ranging from 6 (0800–1400) to 18 (1400–0800) hours. Traps were closed between Friday at 1400 and Sunday at 0800, then remained open until the traps were emptied on Monday at 0800 for a 24-hour exposure time. Each time that the deterrent-integrated trap contents were processed, the data were recorded as a deterrent or control treatment. During the sorting process, fishes were identified to species and counted by Royal Botanical Gardens technicians. During deterrent trials, CO_2_ was pumped into the water continuously, with carbonation starting 20 minutes prior to the trap opening and running until the trap was closed for processing. During the 30 minutes of trap processing, CO_2_ concentrations would return to ambient levels and we reset the trap for the next ambient trial.

## Deterrent stimulus

**Figure 2:**
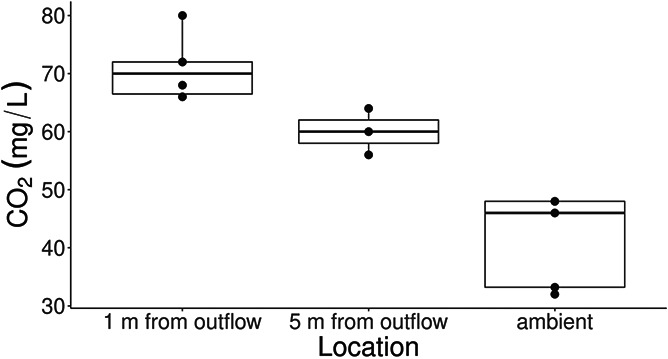
CO_2_ concentrations measured at 1 and 5 m from the CO_2_ deterrent-integrated fishway trap.

A simple CO_2_ deterrent was deployed at the Royal Botanical Gardens fishway and integrated within the centremost trap, which historically had the greatest ambient common carp catch rate (RBG, unpublished data). A submersible pump with a check valve moved wetland water through polyethylene pipe (diameter, 2.54 cm) into an injection manifold (Figure 1). CO_2_ gas was pumped from a liquid CO_2_ dewar (CD MLC230-350; Praxair) into the injection manifold at 10 psi, where the gas line ended with two terminal aerators (10 × 10 ×5 cm) secured inside the manifold. The manifold was a 38 × 28 × 15 cm ABS junction box plumbed to receive the incoming water and gas lines and the outgoing carbonated water line. Aerated CO_2_ bubbles resisted the downward flow of water, increasing bubble/water interface time within the water line. The mixing of CO_2_ and water was assisted with two 25-mm static mixers placed directly below, and ~10 m downline, from the manifold. Inline waterflow downstream of the second static mixer held a CO_2_ concentration of ~400 mg/l and a flow rate of 240 ml/s. The line of carbonated water was then distributed equally among four hoses with a four-way garden hose manifold, where each hose distributed carbonated water to a different region of the modified fishway trap (dimensions, 1.2 × 0.6 m) (Figure 1). The carbonated water maintained a CO_2_ plume directly adjacent to the fishway trap, with minimal plume disruption caused by wetland waterflow (see Cupp *et al.,* 2018) (flowrate at trap, 0.016 ± 0.025 m/s; mean ± standard deviation). CO_2_ concentrations 1 m downstream of the fishway trap and outflow apparatus were 70 ± 5 mg/l (mean ± standard deviation) (Figure 2). Such CO_2_ concentrations have been observed to produce consistent avoidance responses in bigheaded carps in outdoor arenas (Cupp *et al.,* 2016).

## Experimental constraints

CO_2_ mapping was limited due to equipment vandalism inside the locked, gate-secured field site. Underwater CO_2_ probes (eosGP; eosense; Dartmouth, Nova Scotia) were deployed to continuously log CO_2_ ppm throughout the field season. In June 2019, the CO_2_ probes were vandalized while other equipment such as the data-logging laptop and Jon boat used to access the site were stolen. Backed-up data from the underwater CO_2_ probes did not substantially overlap with experimental treatments. Therefore, CO_2_ concentrations reported here were manually calculated with titrations (Hach Company, Loveland, CO, USA; Titrator Model 16 900; CO_2_ Kit No. 2272700 and Total Alkalinity Kit No. 2271900) conducted on water samples from the remaining trials at distances 1 and 5 m downstream of the deterrent-integrated trap, and at a depth of 0.6 m. CO_2_ titrations were collected immediately after samples were collected.

## Statistical analysis

To determine if fish avoided the CO_2_ deterrent, catch abundance for the deterrent-integrated trap of the fishway was analysed in a general linear model. Catch abundance was offset by log-transformed trap exposure time to calculate catch rate. Catch abundances were right-skewed and zero-inflated, so abundance was fit with a Poisson distribution and a log link function. We included species identity and treatment levels as fixed effects that were allowed to interact. Dispersal into the wetland is seasonal and species specific (Bzonek *et al.,* 2021), so we included date as a covariate that was allowed to interact with species identity. We tested for a phylogenetic signal in the species-specific response to the deterrent (Bzonek *et al.,* 2021). Branch lengths and phylogenetic relationships for the species in this study (excluding the common carp x goldfish hybrid *C. carpio x Carassius auratus*) were obtained by pruning the species tree from Rabosky *et al.* (2018). We used the phylosig function in the phytools package (Revell, 2012) to test for a phylogenetic signal in the percent change in catch rates across species. The statistical significance of the phylogenetic signal was evaluated using Pagel’s λ and Blomberg’s K indices of trait evolution. All statistical analyses were performed in R (version 4.0.2; R Foundation for Statistical Computing, Vienna).

## Results

**Figure 3:**
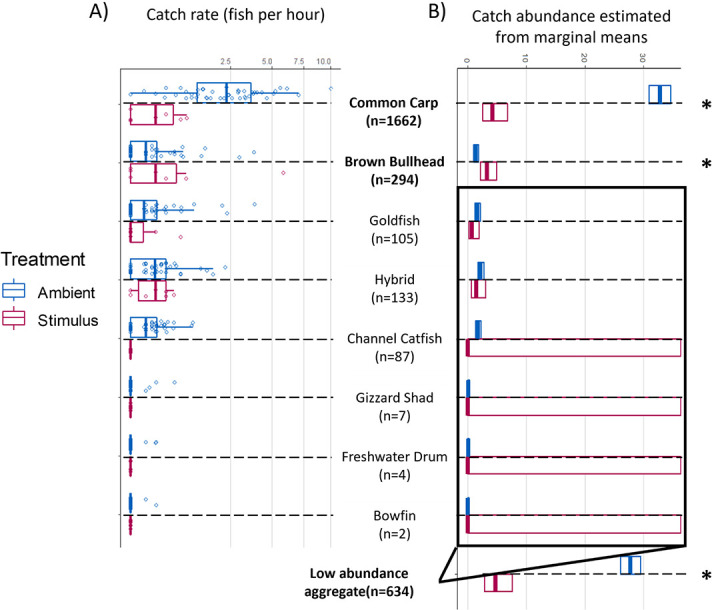
Catch rate of wetland fish species exposed to ambient and deterrent CO_2_ treatments at the Royal Botanical Gardens fishway. (**A**) Raw catch rates of fishes caught in a total abundance than > 1 individual. Each point displays the catch rate of an individual trial, with the boxplots indicating the 25th percentile, median and 75th percentile. (**B**) Estimated marginal mean catch abundance ±95% confidence interval of each species and treatment. The marginal means were estimated from the general linear model reported in the results using the R package ‘emmeans’. The predicted catch abundances of each species were allowed to independently vary across time within the general linear model, so the marginal means presented here were predicted for the median date of the study, 13 June 2019. The catch abundance of each species in each trial was also standardized across trap exposure time; therefore, the catch abundances were predicted for the median trap exposure time of 12.6 hours. Species are indicated between the panels, with the total catch per species indicated in brackets. An estimated marginal mean was also predicted for the aggregation of all low abundance species (150 per species). Catch rates between each treatment were compared for each species in a pairwise *post hoc* Tukey–Kramer test. Ambient and deterrent catch rates significantly differed for common carp, brown bullhead and the aggregation of all low abundance species. Large confidence intervals can be observed for the low abundance species during the deterrent treatment.

**Figure 4:**
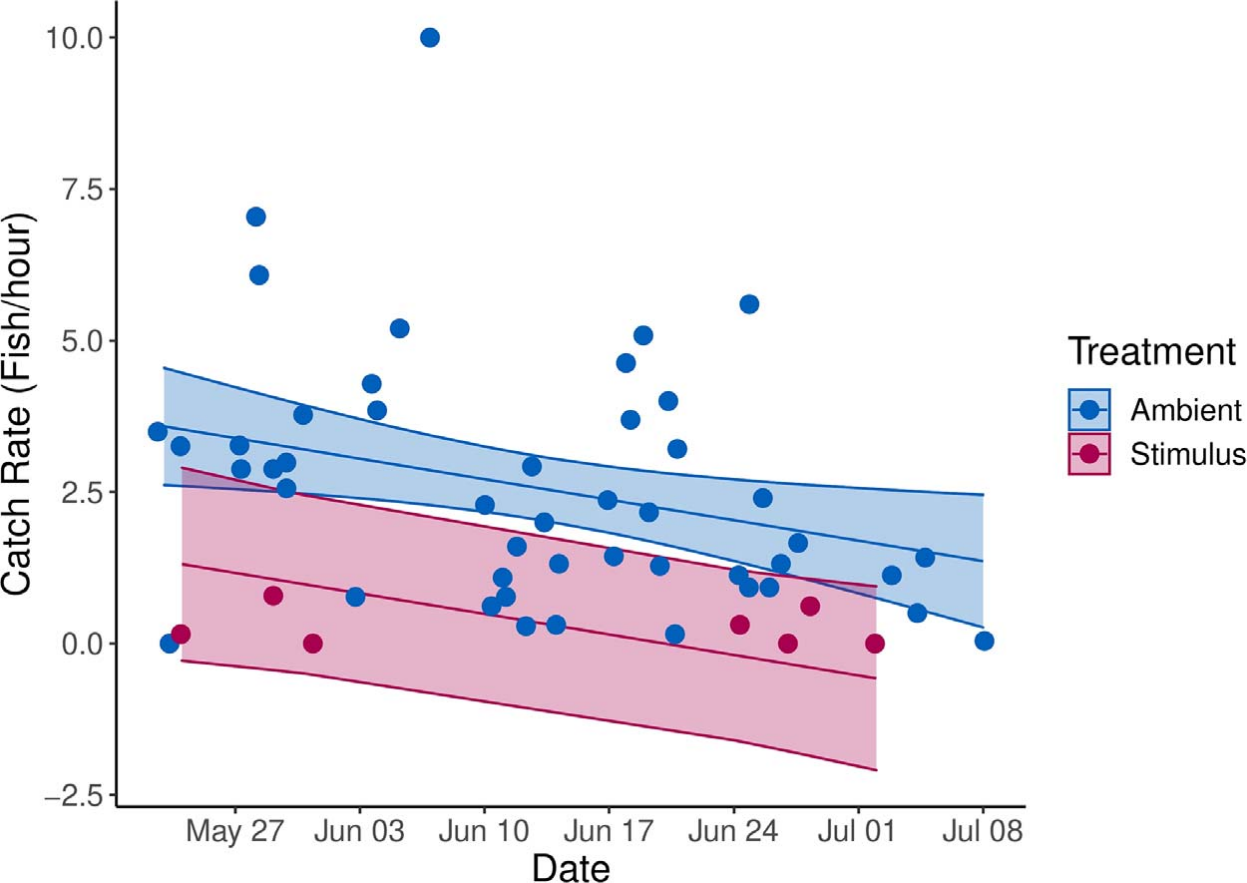
Catch rate (fish/hour) of common carp across time for deterrent and ambient treatments. Missing data for deterrent treatments (1–20 June) is the result of an empty dewar of liquid CO_2_ that could not be exchanged due to flooding. The ribbons indicate the predicted catch rate ±95% confidence interval for each treatment.

Over 2000 fishes, representing 13 species, were captured within the deterrent-integrated trap between 23 May and 8 July 2019 (Table 1). Common carp was the most abundant species, representing ~70% of the individuals captured, with a total catch of 1662 individuals. Brown bullhead (*Ameiurus nebulosus)* was also abundant, with 294 individuals captured throughout the field season. The remaining 11 species represented a catch abundance of 338 individuals, with goldfish (*C. auratus*) and common carp x goldfish hybrids captured in abundances greater than 100 individuals (Figure 3). Common carp catch rates in the deterrent-integrated traps were significantly lower during deterrent activation (Table 2). Catch rates decreased from 2.56 individuals per hour during control trials to 0.26 individuals per hour during deterrent trials (Figure 4). There was an increase in catch rates for brown bullhead, from a control catch rate of 0.31 individuals per hour to a stimulus catch rate of 1.01 individuals per hour. There were no significant statistical differences in catch rates between the control and stimulus treatments for the remaining, less abundant, species. However, when the catch rates of less abundant species were aggregated there was a significant decrease in catch rates, from the control catch rate of 0.77 fish per hour to stimulus catch rate of 0.31 fish per hour. No phylogenetic signal was observed in the species-specific avoidance responses of all regularly captured fishes (Pagel’s λ < 0.01, Blomberg’s K = 0.41, *P* < 0.05).

**Table 1:** Catch data for fishes caught in the CO_2_ deterrent-integrated trap at the Royal Botanical Gardens fishway, 23 May to 8 July 2019

Species scientific name	Common name	Ambient catch rate (fish/hour)	Deterrent catch rate (fish/hour)	Total abundance	Percent change
*C. carpio*	common carp	2.565	0.267	1662	−90%
*A. nebulosus*	brown bullhead	0.308	1.009	294	228%
*C. auratus*	goldfish	0.288	0.110	105	−62%
*C. carpio x C. auratus*	common carp x goldfish hybrid	0.289	0.184	133	−36%
*Ictalurus punctatus*	channel catfish	0.144	0.000	87	−100%
*Dorosoma cepedianum*	gizzard shad	0.017	0.000	7	−100%
*Aplodinotus grunniens*	freshwater drum	0.011	0.000	4	−100%
*Micropterus salmoides*	largemouth bass	0.006	0.000	2	NA
*Amia calva*	bowfin	0.005	0.000	2	NA
*Lepomis gibbosus*	pumpkinseed	0.000	0.022	1	NA
*Oncorhyncus mykiss*	rainbow trout	0.003	0.000	1	NA
*Catostomus commersonii*	white sucker	0.003	0.000	1	NA

**Table 2:** Summary table for the generalized linear model and marginal means describing the catch abundances of species within the deterrent-integrated trap

Species scientific name	Species common name	Treatment	Estimate	Lower confidence level	Upper confidence level	Standard error	*Z* score	*P* value
*C. carpio*	common carp	AmbientDeterrent	32.73224.1763	313	357	0.82410520.9132893	9.367	**<.0001**
*A. nebulosus*	brown bullhead	AmbientDeterrent	1.36363.2709	12	25	0.19429010.6154188	−5.272	**<.0001**
Aggregate of species below		AmbientDeterrent	6.24122.6514	62	51	0.36752150.693775	3.232	**0.0012**
*C. auratus*	goldfish	AmbientDeterrent	1.67750.7096	10	22	0.19977880.3258645	1.871	0.0614
*C. carpio x C. auratus*	common carp x goldfish hybrid	AmbientDeterrent	2.21181.4162	21	33	0.2237820.4862134	1.285	0.1989
*Ictalurus punctatus*	channel catfish	AmbientDeterrent	1.76760	10	2Inf	0.18982370.0000088	0.002	0.9982
*Dorosoma cepedianum*	gizzard shad	AmbientDeterrent	0.12970	00	0Inf	0.05368490.0000077	0.002	0.9981
*Aplodinotus grunniens*	freshwater drum	AmbientDeterrent	0.07810	00	0Inf	0.04066110.0000089	0.002	0.9985
*Amia calva*	bowfin	AmbientDeterrent	0.03980	00	0Inf	0.02877760.0000086	0.002	0.9984
*Micropterus salmoides*	largemouth bass	AmbientDeterrent	0.02520	00	0Inf	0.02681440.0000049	0.002	0.998
*Lepomis gibbosus*	pumpkinseed	AmbientDeterrent	00.0711	00	Inf46	0.00000060.2051705	−0.006	0.995
*Oncorhyncus mykiss*	rainbow trout	AmbientDeterrent	0.02010	00	0Inf	0.02035040.0000083	0.002	0.9985

The marginal means were estimated from the general linear model reported in the results using the R package ‘emmeans’. The predicted catch abundances of each species were allowed to independently vary across time within the general linear model, so the marginal means presented here were predicted for the median date of the study, 13 June 2019. The catch abundance of each species in each trial was also standardized across trap exposure time; therefore, the catch abundances were predicted for the median trap exposure time of 12.6 hours. Catch rates between each treatment were compared for each species in a pairwise *post hoc* Tukey–Kramer test. An estimated marginal mean was also predicted for the aggregation of all low abundance species (per species)

## Discussion

The CO_2_ deterrent produced a strong avoidance response in the target species, common carp, by decreasing catch rates to 10% of their ambient rate. When aggregated, low-abundance species also significantly avoided the deterrent, but abundant brown bullhead did not. Unlike a comparable acoustic deterrent (Bzonek *et al.,* 2021), fishes did not express a phylogenetic signal in avoidance responses. This study exposed several practical challenges relevant to deterrent deployment such as stochastic flooding and equipment vandalism. The inline CO_2_ deterrent used here performed well and may be suitable for continued small-scale, temporary deterrent uses in low flow areas. The results of this proof-of-concept field deterrent are promising for the continued development and deployment of non-physical CO_2_ fish deterrents.

## Avoidance response of target species and greater wetland fish community

Common carp was effectively deterred by the CO_2_ plume and catch rate decreased by 90%, far greater than the 16% decrease caused by the acoustic deterrent in Bzonek *et al.* (2021). A 10-fold decrease in common carp wetland immigration rates could reduce population growth rates if the reduced adult immigration was coupled with wetland harvest (Caskenette *et al.,* 2018). Such harvest is regularly conducted at Cootes Paradise (Lougheed *et al.,* 2004), indicating that, in the context of common carp exclusion at this specific site, a permanent CO_2_ deterrent could have been a viable alternative to a physical barrier. Common carp exclusion can be an effective tool in wetland restoration (Lougheed*et al.,* 1998), and the development of non-structural deterrents may provide greater flexibility for management efforts when physical barriers are not feasible (Noatch and Suski, 2012).

Other species were also affected by the deterrent. Brown bullhead was captured in greater abundance when the deterrent was activated. However, deterrent treatment catch rates for this species were driven by rare, high-abundance trials, including one event where 35 individuals were captured over a 6-hour exposure time. When this large-group outlier is removed, brown bullhead catch rates also decreased with deterrent activation. It is currently unknown whether brown bullhead avoidance responses are affected by group size. Tucker and Suski (2019) found that, for Bluegill (*Lepomis macrochirus*), groups of fish avoided CO_2_ to a greater extent than individuals in isolation, as groups were likely less vulnerable to habituation, learning or inter-individual variation. Bzonek *et al.* (2021) found that group size had no impact on common carp avoidance to an acoustic deterrent. An alternate explanation may be that ictalurids have altered sensitivity, tolerance or behavioural responses to CO_2_ (see Caprio *et al.,* 2014). The 11 remaining fish species represented 14% of the total catch. The catch rates of these species had generally decreased during the deterrent treatment (Table 1), but not significantly. When the catches of all low-abundance species were aggregated, there was a significant decrease in catch rate during the deterrent treatment. Furthermore, closely related species were no more likely to respond similarly to each other than more distantly related species. This result occurred because all species, except brown bullhead, exhibited general avoidance.

Species specificity in deterrent responses may aid in mitigating the ecological impacts of deterrents on non-target species. In contrast to the general avoidance to the CO_2_ deterrent used in this study, an acoustic deterrent deployed at the same site produced a greater avoidance response in ostariophysian fishes than in non-ostariophysian fishes (Bzonek*et al.,* 2021). However, our results indicate that CO_2_ deterrents are likely to alter the dispersal of both target and non-target fish species. Watershed connectivity is a critical factor for many ecological processes (Crook *et al.,* 2015), and the benefits of preventing range expansions of invasive fishes should be balanced against the consequences of limiting the movement of native species. Fausch *et al.* (2009) proposed a conceptual framework for balancing the benefits of invasive species barrier against the costs of population isolation incurred by limiting watershed connectivity. Care should be taken to ensure CO_2_ deterrents are not deployed at sites critical to native fish movement. Sites where naturally isolated waterbodies have been connected through human alteration (e.g. canals, locks) may be ideal sites for deterrent deployment, as the loss of connectivity should have lower impacts on native species.

## Deterrent stimulus

We used CO_2_ as a non-physical deterrent stimulus to prevent common carp entry into a wetland. Such CO_2_ deterrents have shown consistent success in producing avoidance responses in target species (Donaldson *et al.,* 2016; Cupp *et al.,* 2016, 2018; Schneider *et al.,* 2018). This study did not isolate the component of the CO_2_ stimulus to which fishes were responding. Fishes may have avoided the deterrent due to the elevated CO_2_ concentrations. Alternatively, fish may have been avoiding a resultant decrease in O_2_ and pH caused by the elevated CO_2_ (Gilmour, 2001; Perry and Gilmour, 2002). However, we are not certain if either O_2_ or pH decreased in our study to a level that would cause deterrence and recommend future studies monitor both environmental variables. Determining the specific impetus for deterrent avoidance may become important when optimizing the deterrent stimuli but was not critical when establishing proof-of-concept for the deterrent in the field. If the avoidance found in this study was caused by the indirect effects of CO_2_, the stimulus would still be suitable for deterrent use as it is relatively inexpensive, safe to store/transport and effective at altering the water chemistry of the immediate environment.

Additional factors contributed to the suitability of CO_2_ as a deterrent. Water flow across the deployment site was generally static, and ambient CO_2_ concentrations were relatively high. Flow velocity and water discharge have a direct impact on plume development, as moving water carries CO_2_ with it. Our small-scale CO_2_ deterrent would have been less effective at sites with greater water discharge, as flow rates as low as 0.3 m/s have been observed to reduce plume concentrations by 80% elsewhere (Cupp *et al.,* 2018). The relatively high ambient CO_2_ concentrations in Cootes Paradise may have also aided in reaching our target concentration of 60 mg/l (Figure 2). Finally, local or federal permit requirements can be an important factor when considering the deployment of CO_2_ deterrents. For example, CO_2_ has been registered as a pesticide for use as a deterrent for bigheaded carps (*Hypothalmichthyes* spp.) in the USA (Suski, 2020).

## Deterrent design

The strong common carp avoidance response produced by a low-magnitude, temporary CO_2_ deterrent is promising for the development of dedicated, large-scale deterrents. Our deterrent maintained a CO_2_ plume with a 5-m radius and a concentration of ~60 mg/l. This concentration is sufficient to produce an avoidance response in most individuals; however, a higher concentration may be necessary to elicit sufficient avoidance in an unresponsive subset of the population. Large-scale, dedicated deterrents can achieve CO_2_ plumes with higher concentrations than seen here to surpass inter-individual variation in CO_2_ tolerances (Zolper, Cupp, and Smith, 2018). Stronger CO_2_ deterrents may also transition from a behavioural deterrent to a physiological deterrent by surpassing an individual’s tolerance to hypercarbia and induces equilibrium loss (Suski, 2020).

Our inline CO_2_ delivery system operated more efficiently than the terminal-diffuser systems used in comparable outdoor CO_2_ deterrents (Cupp *et al.,* 2016, 2018). When CO_2_ is extruded though porous media at the target site, the resultant bubbles quickly travel through the water column and off-gas into the atmosphere at the surface, dramatically limiting the CO_2_ mass transfer within the water column (see Li *et al.,* 2019). With our design, CO_2_ was diffused into a water line to increase the exposure time of the gas-water interface before the mixture was released at the terminal site. We achieved CO_2 (aq)_ concentrations of ~400 mg/l within our lines, which allowed for the rapid development of CO_2_ plumes at the target site. Our plume concentrations reached 60 mg/l in less than 1 hour, while terminal diffuser systems have required 4–6 hours (Cupp *et al.,* 2016) or >8 hours (Cupp *et al.,* 2018) to produce similar concentrations. Current outdoor deterrent studies have utilized simple deployment designs constructed with relatively common equipment (Cupp *et al.,* 2018). Permanent, large-scale CO_2_ deterrents will require more complex and efficient delivery systems with specialized equipment and access to large volumes of CO_2_. Such deterrents are currently being assessed (Zolper *et al.,* 2018). The deterrent system described here is more suitable for temporary, low flow, small-scale studies where funding, development and operation are limiting factors in deterrent design.

Small-scale CO_2_ deterrents face a number of practical challenges such as the duration of equipment activation and the lack of robust infrastructure. The field site had trail access, but stochastic flooding closed the trail, eliminating vehicle access and conventional gas-canister exchange. Additionally, although the site perimeter was secured, the location was remote and vulnerable to vandalization. Large CO_2_ deterrents will require secure sites with greater infrastructural access to ensure uninterrupted access and permanent CO_2_ storage.

## Conclusion

Non-structural deterrents have been proposed as a means to control the range expansion of invasive fishes without impeding human transport. Before non-structural deterrents can be successfully implemented to halt the range expansions of invasive species, research must address the uncertainties of scaling laboratory-level successes towards field applications (Cupp *et al.,* 2018). This pilot field study demonstrates that an inexpensive CO_2_ deterrent constructed with readily available equipment can produce a robust avoidance response in common carp, a species of significant management concern. These findings support the continued development of more permanent CO_2_ deterrents. Deterrent design should move away from terminal-diffuser systems, which are slower to develop CO_2_ plumes, and deterrent managers should expect a broad avoidance response to CO_2_ plumes from most wetland fishes.

## Funding

This research was funded by the Fisheries and Oceans Canada Asian Carp Program.

## Data Availability

The data underlying this article are available in the online supplementary material.

## Supplementary Material

supplementary_coac021
