# Supplementary material for: Wetland fishes avoid a carbon dioxide deterrent deployed in the field
Source: Conserv Physiol. 2022 May 6;10(1):coac021. doi: 10.1093/conphys/coac021 (PMC9109721; doi:10.1093/conphys/coac021)
Supplement: supplementary_coac021 [file supplementary_coac021.zip › Bzonek_SupplementaryMaterials.docx]

1. **Supplementary Materials:**

Supplementary Table 1: Summary table for generalized linear model describing the catch rate of various species within the deterrent-integrated trap across date and treatment. Incidence rate ratios, standard error, confidence intervals, z statistic and p-values are included.

| Predictors | Incidence Rate Ratios | std. Error | CI | Statistic | p |
| --- | --- | --- | --- | --- | --- |
| (Intercept) | 276.79 | 0.69 | 71.97 – 1064.44 | 8.2 | <0.001 |
| Species [BB] | 0 | 1.53 | 0.00 – 0.00 | -18.43 | <0.001 |
| Species [BW] | 0 | 8.86 | 0.00 – 28180.50 | -0.81 | 0.42 |
| Species [CC] | 0.58 | 0.75 | 0.13 – 2.52 | -0.73 | 0.465 |
| Species [CT] | 0 | 1.51 | 0.00 – 0.02 | -4.44 | <0.001 |
| Species [FD] | 0 | 6.48 | 0.00 – 1.10 | -1.95 | 0.051 |
| Species [GF] | 6.62 | 1.48 | 0.36 – 122.25 | 1.27 | 0.203 |
| Species [GS] | 0.04 | 4.9 | 0.00 – 658.73 | -0.64 | 0.522 |
| Species [Hyb] | 2.11 | 1.33 | 0.16 – 28.69 | 0.56 | 0.573 |
| Species [LMB] | 13.28 | 10.84 | 0.00 – 23568614865.60 | 0.24 | 0.811 |
| Species [PS] | 0 | 431.35 | 0.00 – Inf | -0.11 | 0.912 |
| Species [RT] | 0 | 12.48 | 0.00 – 10763948.84 | -0.67 | 0.505 |
| JDate | 0.96 | 0 | 0.95 – 0.97 | -8.99 | <0.001 |
| Treatment [Stimulus] | 0.42 | 0.26 | 0.25 – 0.71 | -3.23 | 0.001 |
| Species [BB] * JDate | 1.18 | 0.01 | 1.16 – 1.20 | 18.72 | <0.001 |
| Species [BW] * JDate | 1.01 | 0.05 | 0.91 – 1.13 | 0.23 | 0.816 |
| Species [CC] * JDate | 1.01 | 0 | 1.00 – 1.02 | 2.87 | 0.004 |
| Species [CT] * JDate | 1.03 | 0.01 | 1.02 – 1.05 | 3.61 | <0.001 |
| Species [FD] * JDate | 1.05 | 0.04 | 0.97 – 1.13 | 1.31 | 0.192 |
| Species [GF] * JDate | 0.98 | 0.01 | 0.96 – 1.00 | -2.07 | 0.038 |
| Species [GS] * JDate | 1 | 0.03 | 0.94 – 1.06 | -0.15 | 0.884 |
| Species [Hyb] * JDate | 0.99 | 0.01 | 0.97 – 1.01 | -1.3 | 0.194 |
| Species [LMB] * JDate | 0.95 | 0.07 | 0.83 – 1.09 | -0.7 | 0.486 |
| Species [PS] * JDate | 1.18 | 0.18 | 0.82 – 1.69 | 0.91 | 0.363 |
| Species [RT] * JDate | 1.02 | 0.08 | 0.87 – 1.18 | 0.2 | 0.838 |
| Species [BB] * Treatment [Stimulus] | 5.65 | 0.31 | 3.06 – 10.43 | 5.54 | <0.001 |
| Species [BW] * Treatment [Stimulus] | 0 | 1150.76 | 0.00 – Inf | -0.01 | 0.991 |
| Species [CC] * Treatment [Stimulus] | 0.3 | 0.34 | 0.15 – 0.59 | -3.49 | <0.001 |
| Species [CT] * Treatment [Stimulus] | 0 | 1254.36 | 0.00 – Inf | -0.01 | 0.99 |
| Species [FD] * Treatment [Stimulus] | 0 | 1274.98 | 0.00 – Inf | -0.01 | 0.992 |
| Species [GF] * Treatment [Stimulus] | 1 | 0.53 | 0.35 – 2.82 | -0.01 | 0.994 |
| Species [GS] * Treatment [Stimulus] | 0 | 1092.73 | 0.00 – Inf | -0.01 | 0.99 |
| Species [Hyb] * Treatment [Stimulus] | 1.51 | 0.44 | 0.64 – 3.55 | 0.94 | 0.347 |
| Species [LMB] * Treatment [Stimulus] | 0 | 951.19 | 0.00 – Inf | -0.01 | 0.989 |
| Species [PS] * Treatment [Stimulus] | 16754553 | 430.07 | 0.00 – Inf | 0.04 | 0.969 |
| Species [RT] * Treatment [Stimulus] | 0 | 1206.09 | 0.00 – Inf | -0.01 | 0.992 |
| Observations | 648 |  |  |  |  |
| R2 Nagelkerke | 1 |  |  |  |  |
